# First-line HIV treatment failures in non-B subtypes and recombinants: a cross-sectional analysis of multiple populations in Uganda

**DOI:** 10.1186/s12981-019-0218-2

**Published:** 2019-01-22

**Authors:** Art F. Y. Poon, Emmanuel Ndashimye, Mariano Avino, Richard Gibson, Cissy Kityo, Fred Kyeyune, Immaculate Nankya, Miguel E. Quiñones-Mateu, Eric J. ARTS, The Ugandan Drug Resistance Study Team, Nicholas I. Paton, Sarah Walker, Anne Hoppe, Tobias F. Rinke de Wit, Kim C. E. Sigaloff, Raph Hamers, T. Sonia Boender, Ragna S. Boerma, Pascale Ondoa, Marloes Nijboer, Stefanie Kroeze, Seth Inzaule, Cissy Kityo Mutuluuza, Alani Sulaimon Akanmu

**Affiliations:** 1 Department of Pathology and Laboratory Medicine, Health Sciences Addition, H422, Western University, London, ON N6G 2R5 Canada; 2 Department of Microbiology and Immunology, Western University, London, Canada; 3 Department of Applied Mathematics, Western University, London, Canada; 4 Joint Clinical Research Centre, Kampala, Uganda; 5 Center for AIDS Research Uganda Laboratories, Joint Clinical Research Centre, Kampala, Uganda; 6 Department of Molecular Biology and Microbiology, Case Western Reserve University, Cleveland, OH USA; 7 Department of Medicine, Case Western Reserve University, Cleveland, OH USA; 8 Department of Pathology, Case Western Reserve University, Cleveland, OH USA

**Keywords:** HIV-1 subtypes, Sub-Saharan Africa, Drug resistance, Treatment failure, Recombination

## Abstract

**Background:**

Our understanding of HIV-1 and antiretroviral treatment (ART) is strongly biased towards subtype B, the predominant subtype in North America and western Europe. Efforts to characterize the response to first-line treatments in other HIV-1 subtypes have been hindered by the availability of large study cohorts in resource-limited settings. To maximize our statistical power, we combined HIV-1 sequence and clinical data from every available study population associated with the Joint Clinical Research Centre (JCRC) in Uganda. These records were combined with contemporaneous ART-naive records from Uganda in the Stanford HIVdb database.

**Methods:**

Treatment failures were defined by the presence of HIV genotype records with sample collection dates after the ART start dates in the JCRC database. Drug resistances were predicted by the Stanford HIVdb algorithm, and HIV subtype classification and recombination detection was performed with SCUEAL. We used Bayesian network analysis to evaluate associations between drug exposures and subtypes, and binomial regression for associations with recombination.

**Results:**

This is the largest database of first-line treatment failures ($n=1724$) in Uganda to date, with a predicted statistical power of 80% to detect subtype associations at an odds ratio of $\ge 1.2$. In the subset where drug regimen data were available, we observed that use of 3TC was associated with a higher rate of first line treatment failure, whereas regimens containing AZT and TDF were associated with reduced rates of failure. In the complete database, we found limited evidence of associations between HIV-1 subtypes and treatment failure, with the exception of a significantly lower frequency of failures among A/D recombinants that comprised about 7% of the population. First-line treatment failure was significantly associated with reduced numbers of recombination breakpoints across subtypes.

**Conclusions:**

Expanding access to first-line ART should confer the anticipated public health benefits in Uganda, despite known differences in the pathogenesis of HIV-1 subtypes. Furthermore, the impact of ART may actually be enhanced by frequent inter-subtype recombination in this region.

**Electronic supplementary material:**

The online version of this article (10.1186/s12981-019-0218-2) contains supplementary material, which is available to authorized users.

## Background

East Africa was one of the first regions in the world to experience high rates of HIV infection [1]. In 1990, for instance, some antenatal clinics in Uganda recorded adult HIV prevalences among women exceeding 30% [2]. Currently, the adult prevalence of HIV in Uganda is about 7.1% [3]. Increasing the coverage of combination antiretroviral therapy in Uganda is a crucial public health objective to not only reduce HIV-related morbidity and mortality, but also to prevent the onward transmission of HIV by reducing plasma viral loads [4, 5]. However, the enormous genetic diversity of the HIV-1 subtypes has been a persistent concern for antiretroviral treatment, especially in low- and middle-income countries like Uganda with multiple prevalent subtypes [6]. Antiretroviral drugs have generally been developed and tested on HIV subtype B [7], which is the predominant subtype in North America and western Europe [8]. HIV-1 infections in Uganda are predominated by subtypes A and D [9], with a low frequency of subtype C that is the predominant subtype in southern Africa. In addition, recombinants of subtypes A and D have historically been observed in about 10% to 30% of HIV infections sampled in Uganda [10, 11]. Based on a phylogenetic analysis of dated HIV sequences, subtype A likely migrated into Uganda around the 1950s before subtype D entered about a decade afterwards [12]. To date, subtype A HIV-1 infections are more prevalent in the east and north regions of Uganda, while subtype D dominates in the west and south of this small country [13].

There is accumulating evidence of clinically-significant differences among the HIV-1 subtypes. For example, multiple studies have observed that subtype D is associated with a faster rate of disease progression relative to subtype A in the absence of treatment [14–16]. Our previous 15-year natural history study of HIV disease progression confirmed these observations, but also described a significantly slower rate of disease progression in subtype C HIV-1 infected individuals over those infected with subtype A or D [16, 17]. Previous studies of serodiscordant couples have also reported that subtype D has a lower transmission rate than subtype A [18], which is consistent with a decline in the overall prevalence of subtype D in the region [19]. In addition, HIV subtype variation can have an impact on the emergence of drug resistance mutations. For example, the HIV-1 RT mutation K65R emerges more rapidly in subtype C infections due to variation among subtypes in a homopolymeric region that interrupts reverse transcription and induces a higher rate of base misincorporation [20].

Evaluating the clinical significance of HIV non-B subtypes in the context of antiretroviral treatment (ART) remains a significant challenge and few studies have the necessary sample sizes in resource-limited settings, let alone in any specific region. In a recent systematic review of switching to second-line treatments in sub-Saharan Africa [21], the estimated incidence averaged about 2.6 first-line treatment failures per 100 person-years and ranged between 2 to 5 per 100 person-years. Consequently, prospectively enrolling patients on first-line treatment can severely limit the expected number of treatment failures in the sample population. In this study, we have combined HIV sequence and clinical data associated with first-line treatment failures from multiple study cohorts and clinical sites based in Uganda ($n=1724$). These data were supplemented with drug-naive (baseline) HIV sequence data from the same clinical sites ($n=968$) and location-time matched records from the Stanford HIV drug resistance database ($n=1462$) [22] for a combined total of 4154 patient records. To date, this represents the largest retrospective multi-site analysis of HIV first-line treatment failures in Uganda, and likely in all of sub-Saharan Africa. The primary objective of our study was to assess the clinical significance of HIV-1 subtypes in the context of first-line treatment failure. Results from our investigation then motivated a deeper analysis on the impact of frequent inter-subtype recombination in this region where both subtypes A and D are prevalent. Here we report evidence that first-line treatment failure in Uganda is generally more strongly associated with drug regimens than HIV-1 subtypes. By expanding our database, however, we observe that A/D recombinants are significantly less associated with first-line treatment failure, and that this effect is more pronounced with an increasing number of putative recombination breakpoints in HIV-1 *pol*.

## Methods

### Data collection

Anonymized HIV genotypes and clinical data records were collected from multiple clinical sites and cohort studies (Additional file 1: Table S1). Samples from the Europe–Africa Research Network for Evaluation of Second-line Therapy (EARNEST) trial [23] were collected under a protocol approved by the research ethics committee at University College London, with written informed consent provided by patients, or by caregivers for patients under 18 years of age. The sample collection protocol for the Hormonal Contraception and HIV-1 Genital Shedding and Disease Progression among Women with Primary HIV Infection (GS) study [16, 24] was approved by the institutional review boards of the collaborating institutions, and all participants provided informed consent. Samples from the Pan-African Studies to Evaluate Resistance (2008, PASER) network [25] were collected under a protocol approved by research ethics committees at the collaborating sites and the Academic Medical Center (AMC) of the University of Amsterdam, and all participants provided written informed consent. Samples from the Joint Clinical Research Centre (JCRC), the main HIV care provider in Uganda, included patient samples from the JCRC clinics (2005–2016, DR) and the Monitoring Antiretroviral Resistance in Children (2010–2011, MARCH) observational cohort study [26] with informed consent provided by parent(s)/guardian(s); the sample collection protocol was approved by the ethical committees at the JCRC and the AMC. Our analysis of these anonymized data was collectively approved by the institutional review board of the JCRC (EM10-07).

Samples collected in countries other than Uganda (e.g., Zimbabwe, Malawi) were excluded from further analyses. First-line treatment failures were defined by the presence of an HIV drug resistance genotype record in the database subsequent to start of treatment, which implied a detectable viral load ($>50$ copies/mL plasma). Drug exposures were recorded as the drug regimen at the start of treatment for baseline samples, and the regimen at the time of virological failure otherwise. The median collection year of samples was 2010, with the earliest sample collected in 2005 and the most recent in 2016; this study was initiated in August 2016.

### Data processing

We used a rules-based record linkage algorithm to associate these sequence-derived data to clinical variables in a separate database, with a customized rule set for the sequence/patient label nomenclature of each study population. This record linkage was determined to be a necessary processing step when initial analyses found discordant patient identifiers between databases that were likely the result of errors during manual data entry. The clinical data included cohort study, region, gender, age at enrollment, plasma viral load and CD4 cell count at baseline, and first-line ART regimen. To augment the number of baseline samples in the data, we merged the sequence database with the genotype-treatment correlation dataset published by the Stanford HIVdb database [22], which we reduced to only drug-naive records collected in Uganda ($n=1462$). Note that our linkage algorithm was applied only to anonymized data collected from clinical sites and cohort studies associated with the JCRC; no linkage was applied to any data from the Stanford database. Since the Stanford HIVdb records spanned a broader range of sample collection dates, we excluded all HIVdb records that were sampled prior to 2005 to ensure that the HIVdb sequences were contemporaneous with samples from our multi-cohort study. We verified that none of the HIVdb sequences duplicated records in the JCRC database. Resistance predictions for HIV sequences were generated with the Stanford HIVdb algorithm [22]. Subtype predictions from nucleotide sequences were obtained using SCUEAL [27] and verified with REGA (version 3.0) [28] and by phylogenetic reconstruction. Complete details on the sequence analysis methods are provided as Additional file 1: Text S1.

### Statistical analysis

All statistical analyses, including generalized linear models (GLMs), were performed in the R computing environment unless noted otherwise. Associations between categorical variables were evaluated using Fisher’s exact tests. We used a log-transformation of plasma viral loads and a cubic root transformation of CD4 cell counts to accommodate the normality assumption of parametric tests. To fit GLMs to the genotypic susceptibility score (GSS) data [29], which are calculated to a resolution of 0.25 units, we rounded these outcomes to the nearest integer and used a binomial logit-link function. Statistical tests were generally reported by 95% confidence intervals; in cases where we reported *P*-values, significance was interpreted at a threshold of $\alpha =0.05$ unless otherwise noted. Cases with missing data were dropped from the respective analyses. A Bayesian network analysis was performed using a custom implementation in HyPhy [30] (see Additional file 1: Text S2).

## Results

### Subtype distribution

**Table 1:** Summary table for HIV-1 baseline and treatment failure samples

Variable	Baseline	%	Failure	%	95% CI
	2430	58	1724	42	
*Gender*					
Male	n/a		208	43.2	
Female	n/a		232	56.8	
Age (years)	n/a		19 (13–39)^a^		
*Region*					
Fort Portal	222	30.5	86	7.5	
Gulu	0	0	18	1.6	
Kabale	0	0	26	2.3	
Kampala	252	34.7	910	79.5	
Mbale	253	34.8	82	7.2	
Mbarara	0	0	22	1.9	
*Drug exposures*					
3TC/AZT/NVP	172	23.8	318	26.0	
3TC/AZT/EFV	163	22.6	196	16.0	
3TC/d4T/NVP	46	6.4	208	17.0	
EFV/FTC/TDF	179	24.8	36	2.9	
3TC/EFV/TDF	0		113	9.2	
Other	161	22.3	351	28.7	
log_10_ viral load	5.2 (4.7–5.9)^a^		4.8 (4.2–5.3)^a^		0.35 to 0.54^b^
CD4 cell count	n/a		111 (35–232)^a^		
*Subtype predictions*					
A	1028	42.3	758	44	0.94 to 1.21^c^
A/D	217	8.9	85	4.9	0.40 to 0.69
C	60	2.5	56	3.2	0.90 to 1.95
D	751	30.9	503	29.2	0.80 to 1.06
Other	374	15.4	322	18.7	1.06 to 1.49
Recombination breakpoints					
0	1634	67.2	1234	71.6	0.02 to 0.31^d^
1	124	5.1	84	4.9	(− 0.20) to (− 0.03)^e^
2	327	13.5	248	14.4	
3	187	7.7	94	5.5	
4	158	6.5	64	3.7	

Table counts do not include cases with missing data on region, drug exposure, viral load or CD4 cell counts

*n/a* indicates that no data were available for the variable and group, *3TC* lamivudine, *AZT* zidovudine, *NVP* nevirapine, *EFV* efavirenz, *d4T* stavudine, *FTC* emtricitabine, *TDF* tenofovir

^a^Numbers correspond to the median and interquartile range (in parentheses). 95% confidence intervals are reported for the following test statistics: ^b^ the difference between the means of two groups from Student’s *t*-test. ^c^ the odds ratio for Fisher’s exact test; ^d^ the difference in treatment failures in log odds of structural zeroes in a zero-inflated Poisson (ZIP) model, and; ^e^ the decrease in the log-transformed number of breakpoints among treatment failures under the ZIP model

**Fig. 1:**
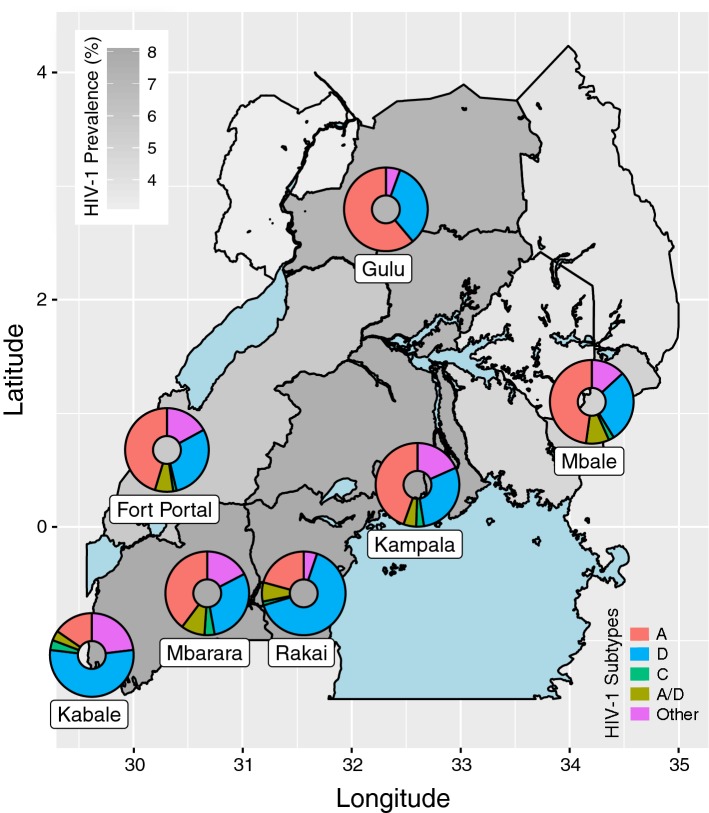
Regional distribution of HIV-1 subtypes in Uganda. The country sub-regions are shaded with respect to the estimated HIV-1 prevalence (%) among adults aged 15–64 from the 2017 Uganda Population-Based HIV Impact Assessment (UPHIA). Subtype frequencies (by colour, see legend) for population centres represented in our database are depicted with ring charts mapped to their geographic locations: Fort Portal ($n=308$); Gulu ($n=18$); Kabale ($n=26$); Kampala ($n=1162$); Mbale ($n=335$); Mbarara ($n=513$); Rakai ($n=135$)

We identified 968 baseline and 1724 first-line treatment failures from the Ugandan study populations, and an additional 1462 drug-naïve samples from Uganda in the Stanford HIVdb database for a total of 4154 samples (Table 1). This sample size is predicted to have sufficient power to detect an association between treatment failure and subtype at an odds ratio of $\sim$1.2 or greater, given an overall 5% prevalence of failure and a subtype frequency of 20% (Additional file 1: Figure S1). Figure 1 displays the distribution of HIV-1 subtypes across regions of Uganda. Overall, HIV-1 subtype A was the most prevalent in our database (43%), followed by subtype D (30%). We also observed a greater frequency of A/D recombinants (7%) than subtype C (3%). About 17% of sequences received ‘other’ subtype/recombinant classifications; the most common variants within this category were unclassified subtype U ($n=116$) and A1/U recombinants ($n=100$). In our subsequent phylogenetic analysis (Additional file 1: Figure S2), subtype U sequences and sequence fragments were placed within or adjacent to the subtype A subtree; hence, they may represent subtype A lineages that are distinct from the subtype reference sequences. We retained the original subtype assignments for the remainder of our analyses. The proportionate agreement between the SCUEAL and REGA algorithms in assigning subtype A, C and D and A/D recombinants was $96.4\%$ (Cohen’s $\kappa =0.94$; Additional file 1: Table S2).

### Treatment failure and drug resistance

**Fig. 2:**
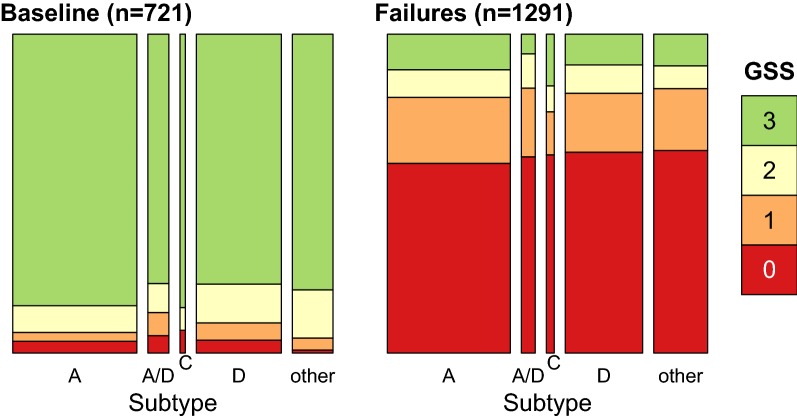
Genotype susceptibility scores (GSS) by group and HIV-1 subtype. GSS was calculated from the Stanford resistance scores and drug regimens; to facilitate visualization, the scores were rounded to the nearest integer and capped at a maximum of 3 (highly susceptible genotype, green). Each set of stacked bars represents the proportion of sequences in each GSS category for a given subtype. The area of each set of stacked bars is proportional to the total number of individuals in each subtype category

We observed significant regional differences in drug exposure: for example, 3TC was more frequently prescribed in Kampala than other regions (Fisher’s exact test, odds ratio $OR=2.45$, 95% CI = [1.95, 3.07]) and EFV was prescribed less frequently ($OR=0.61$ [0.50, 0.75]). To evaluate whether drug resistance patterns were consistent with treatment failures, we calculated the genotypic susceptibility score (GSS) for each individual with a known drug regimen ($n=$ 2012). The GSS can be interpreted as the number of effective drugs in an individual’s regimen, given the genetic makeup of their virus population [29], where drug effectiveness is based on the Stanford HIVdb resistance score. The mean GSS among failure samples (0.77) was significantly lower than the mean among baseline samples (2.71; binomial GLM effect estimate = $-\,1.27$ log odds, 95% CI = [$-\,1.32$, $-\,1.23$]; Fig. 2), which was consistent with the virus populations accumulating resistance mutations in response to each patient’s drug regimen. We found no significant effect of subtype on GSS when this term was added to the model, which implied that patterns of drug resistance in treatment failures were similar across subtypes.

**Fig. 3:**
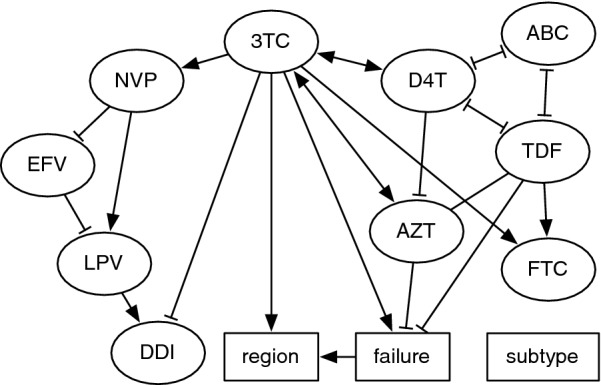
Consensus Bayesian network of antiretroviral (ARV) exposure, region and treatment failure associations. Each node represents a discrete-valued variable from the data. Nodes representing ARVs are labeled with the standard abbreviation. A line (edge) between nodes indicates a conditional dependence between the respective variables with a marginal posterior probability (MPP) exceeding 90%. Triangular arrowheads indicate positive associations, and T-shaped arrowheads indicate negative associations. Edges with a single arrowhead indicate a putative directional effect with MPP > 80%; undirected edges have double arrowheads

Next, we used Markov chain Monte Carlo sampling to fit a Bayesian network model [31] with baseline/failure as a binomial variable to evaluate associations between specific drugs and treatment failure, while accommodating for potential subtype differences, geographic regional differences, and drugs employed in combination therapy (Fig. 3). Convergence between replicate chain samples was accessed with respect to posterior probability using the Gelman-Rubin diagnostic (upper confidence limit = 1.01). The model was fit to the subset of records with complete information about sampling region, subtype classification and drug exposures ($n=1750$). Of this subset, $n=721$ represented baseline records and $n=1029$ represented treatment failures (Table 1). The distribution of edges among nodes representing antiretroviral drugs was consistent with common drug combinations. For example, we obtained well-supported edges connecting the nodes representing the drugs 3TC, AZT and NVP. Our network model suggests that, once regional differences and drug combinations were accounted for, only 3TC was positively associated with treatment failure (marginal posterior probability, $\text {MPP}=0.95$). Similarly, the model predicted that AZT and TDF were negatively associated with failures (both $\text {MPP}=0.91$). Our consensus Bayesian network did not detect any significant associations between HIV-1 subtypes and any other variables in the network, including first-line treatment failures (Fig. 3). This result indicated that any statistical association between first-line treatment failure and HIV-1 subtypes was overwhelmed by the effects of drug exposures. Overall, when accounting for subtype, regional sites in Uganda, and drug regimens, the most significant correlate of first line treatment failure appeared to be the use of 3TC. These results imply that associations between HIV-1 subtypes and first-line treatment failure, if any, were not driven by drug exposure or resistance.

### Clinical differences among HIV-1 subtypes

Plasma viral load (pVL) measurements were available for 579 baseline samples and 962 failure samples. The mean difference in pVL between baseline and failures samples was 0.44 $\log _{10}$ units, and statistically significant (Table 1). There was not significant variation in $\log _{10}$ pVL among subtypes at baseline (ANOVA, $P=0.85$), but marginally significant variation in failure samples ($P=0.04$; Additional file 1: Figure S3). Within treatment failures, individuals with subtype C infections tended to have lower pVL than the other subtypes (mean difference = $-\,0.38 \log _{10}$ units; Student’s *t* test, 95% CI = [$-\,0.75$, $-\,0.02$]); this result is consistent with previous work in this population [16]. CD4 cell counts were only available for 141 failure samples, and no counts were available for baseline samples (Table 1). We observed no significant variation in CD4 among subtypes (ANOVA, $P=0.09$).

### Fewer recombinants among treatment failures

Table 1 and Additional file 1: Figure S4 depict the overall distribution of HIV-1 subtypes between baseline and failure samples in the entire database ($n=4154$). We found that SCUEAL-defined A/D recombinants ($n=302$) were significantly less frequent in failure samples (Fisher’s exact test, odds ratio $OR=0.53$, 95% CI = [0.40, 0.69]). A similar association was obtained with REGA-defined A/D recombinants ($n=284$, $OR=0.34$ [0.25, 0.46]); for brevity, the remainder of our analysis will utilize the SCUEAL predictions. To assess whether this effect was caused by combining the Uganda clinical and study population data with published sequences from the Stanford HIVdb database, we repeated our analysis excluding the HIVdb data. The frequency of A/D recombinants remained significantly lower among failure samples in this reduced data set ($OR=0.58$ [0.41, 0.80]). We also observed that sequences classified by SCUEAL into the ‘other’ category, which comprises subtype U (unclassified) and inter-subtype recombinants other than A/D, were significantly more likely to occur in failure samples ($OR=1.3$ [1.07, 1.49]; Additional file 1: Figure S4). However, the reproducibility of classifying sequences into the ‘other’ category was low (Additional file 1: Table S2). Unexpectedly, subtype U sequences alone were significantly more frequent among failure samples ($OR=2.0$ [1.38, 3.03]); no such association was observed for sequences categorized as subtype A ($OR=1.07$ [0.94, 1.21]). Thus, although the subtype U sequences appear to be evolutionarily related to subtype A (Additional file 1: Figure S2), our data suggest that these unclassified variants may be associated with increased rates of first-line treatment failure.

The number of inferred breakpoints in A/D recombinants was significantly negatively associated with first-line treatment failures (binomial GLM effect estimate = $-\,0.39$ [$-\,0.67$, $-\,0.12$] log odds per breakpoint). For instance, HIV-1 sequences with more than two recombination breakpoints were about 60% less likely to appear in failure samples than expected by chance. This effect was not influenced by sequence length (likelihood-ratio test, $P=0.94$). We also found that the number of breakpoints remained significantly lower in failure samples when we expanded our analysis to the entire database including non-recombinants and other inter-subtype recombinants. Since this distribution included a large class of zero breakpoints, we fit a zero-inflated Poisson model to these counts [32]. We found that treatment failures had a significantly greater chance of carrying a non-recombinant strain (log odds = $+\,0.16$, 95% CI = [0.02, 0.31]) and a significantly reduced number of breakpoints when carrying a recombinant strain (log odds = $-\,0.12$, [$-\,0.2$, $-\,0.03$]; Table 1).

**Fig. 4:**
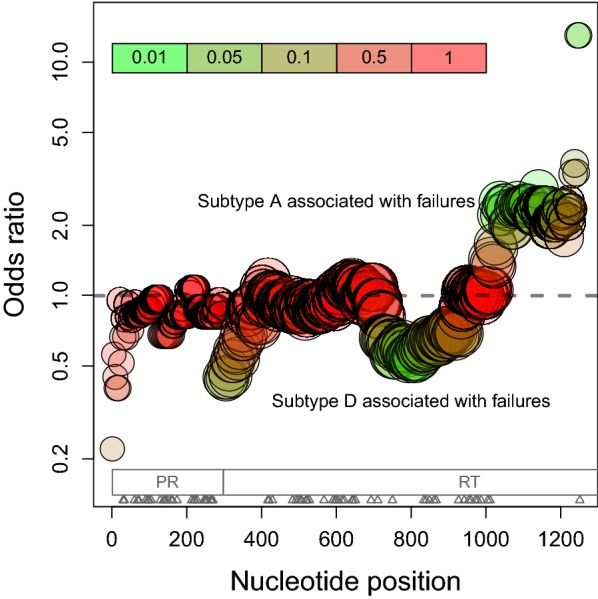
Nucleotide-level associations between HIV-1 subtype and first-line treatment failures in HIV-1 A/D recombinants (*n* = 302). Each circle represents the result of a Fisher’s exact test at a specific nucleotide position in the HIV-1 *pol* reference sequence (*x*-axis). The locations of resistance-associated sites (triangles) within HIV-1 protease (PR) and reverse transcriptase (RT) are indicated at the bottom of the plot region. The area of circles were scaled in proportion to the sample size (range *n* = 79–302) at the respective nucleotide site, due to the varying coverage of partial sequences. Circles were coloured with respect to the *P*-value of each test (see inset legend). To avoid cluttering the plot, we thinned the number of tests to regular intervals of four nucleotides

Since we found a significantly lower frequency of A/D recombinants in the sample population of first-line treatment failures, we further examined associations between HIV-1 recombination and treatment failures. First, we visualized the distribution of inferred recombination breakpoints and assignments of recombinant fragments to HIV-1 subtypes A and D (Additional file 1: Figure S5). This plot implies a complex evolutionary history of A/D recombinants in Uganda, with recombinants arising from multiple events. Next we examined whether particular A/D recombinant fragments were more associated with first-line treatment failures using a series of nucleotide-level association tests along the length of the HIV-1 *pol* sequence. This analysis revealed that associations between subtype A-derived fragments and treatment failures tended to cluster towards the 3′end/C-terminus of RT, just downstream of the resistance associated sites in RT (Fig. 4).

## Discussion

This study represents the largest database of first-line treatment failures in Uganda to date, drawing from every available study cohort associated with the JCRC. In a previous cross-sectional study of a clinical HIV database in Uganda, we reported a statistical association between HIV subtype D infections and treatment failures on second-line and salvage therapies ($n=843$) [33], which are generally associated with higher failure rates than first-line therapies [34]. Furthermore, suboptimal modification of drug regimens in second-line and salvage therapies may allow substantial virus replication to amplify incipient differences among HIV-1 subtypes. In this study, we observed limited evidence for significant variation with respect to genotype susceptibility scores and plasma viral loads among HIV-1 subtypes within the first-line treatment failures. We acknowledge some insurmountable limitations in this retrospective cross-sectional study of all available records of first-line treatment failures in the JCRC databases. For instance, matched baseline and failure samples or duration of treatment were not available for the majority of cases, which would have permitted a longitudinal analysis of these populations. Moreover, our Bayesian network analysis was limited to the $n=1750$ (42%) records with complete information on drug regimens and sampling region.

Examining the marginal distribution of subtypes and failures in the entire database ($n=4154$), we recovered a significant and unexpected negative association between A/D recombinants and first-line treatment failures (Additional file 1: Figure S4). We found no significant association of other inter-subtype recombinants with first-line treatment failures, although the relatively low frequencies of such recombinants in the database limited our power to detect such associations. Inter-subtype HIV-1 recombinants, estimated to comprise roughly 20% of all infections worldwide, are increasingly significant to global public health [35]. The effective rate of recombination in HIV is comparable to the mutation rate [36]. Recombination of divergent subtypes does not necessarily confer a fitness advantage to the virus; for instance, it may disrupt co-adapted combinations of genetic differences unique to the respective subtypes [37]. We note that in the field of HIV-1, the term ‘fitness’ tends to be reserved for the replication of the virus in the absence of treatment. However, variation in viral fitness is biologically and clinically relevant even in the context of treatment, which constitutes a shift in the environment that modulates the fitness of different genotypes. Since a large number of genetic differences are fixed among subtypes, recombination between specific subtypes will predictably generate certain genotype combinations. Hence, even if recombination among subtypes has no net effect on virus fitness, it is reasonable for recombinants of particular subtypes (e.g., A and D) to be, on average, less fit or more fit than their respective parental variants. Several groups have constructed inter-subtype recombinants and performed functional assays or competitive growth experiments in vitro against the parental strains [38, 39]. Overall, the experimental results are equivocal with some recombinants displaying a slight but significant fitness advantage over the parental strains, while other recombinants are less fit [40]. Our results predict that recombination experiments in *pol* between subtypes A and D should tend to produce less fit recombinant viruses on average, and that variation in fitness among recombinants may be predictable from the composition of the recombinant HIV-1 *pol* gene sequence.

Overall, our findings are consistent with findings suggesting rates of disease progression among HIV-1 subtypes does not have major impact on response to treatment [6, 16]. With any difference in treatment regimen, our Bayesian network analysis on a subset of the data indicates that specific drug usage would mask any subtype effect. Only by expanding to the entire database and masking the drug regimens do we detect evidence of reduced failures in patients infected with A/D recombinants. Based on the analyses and models described above, it is remarkable to observe that relatively frequent recombination between the prevalent subtypes A and D may actually promote the effectiveness of first-line treatment regimens in Uganda.

## Additional file

**Additional file 1.** Additional text, figures and tables are provided in support of results in the main text, including methodological details on HIV-1 sequence processing.

## Abbreviations

ART: antiretroviral treatment
JCRC: Joint Clinical Research Centre in Uganda
SCUEAL: Subtype Classification Using Evolutionary Algorithms
EARNEST: Europe-Africa Research Network for Evaluation of Second-line Therapy trial
PASER: Pan-African Studies to Evaluate Resistance network
AMC: Academic Medical Center of the University of Amsterdam
MARCH: Monitoring Antiretroviral Resistance in Children observational cohort study
GS: the Hormonal Contraception and HIV-1 Genital Shedding and Disease Progression among Women with Primary HIV Infection study
DR: clinical samples collected for drug resistance testing at the JCRC
REGA: not an abbreviation, refers to the Rega Institute for Medical Research of the Katholieke Universiteit Leuven
GLM: generalized linear model
GSS: genotypic susceptibility score
3TC: lamivudine
AZT: zidovudine
NVP: nevirapine
EFV: efavirenz
d4T: stavudine
FTC: emtricitabine
TDF: tenofovir
MPP: marginal posterior probability
pVL: plasma viral load
ANOVA: analysis of variance
CI: confidence interval
RT: reverse transcriptase
