# Supplementary material for: First-line HIV treatment failures in non-B subtypes and recombinants: a cross-sectional analysis of multiple populations in Uganda
Source: AIDS Res Ther. 2019 Jan 22;16:3. doi: 10.1186/s12981-019-0218-2 (PMC6343277; doi:10.1186/s12981-019-0218-2)
Supplement: Supplementary file 1 — Additional file 1. Additional text, figures and tables are provided in support of results in the main text, including methodological details on HIV-1 sequence processing. [file 12981_2019_218_MOESM1_ESM.pdf]

Additional File 1

Additional Text

Text S1: Sequence analysis

Sequence data from the Ugandan study populations were processed by the following steps. Multiple HIV genotypes from the same patients, including partial sequences of protease and RT respectively, were identified by parsing the sequence labels with study-specific regular expressions. Plurality consensus sequences at baseline and, if applicable, treatment failure were produced for each patient by using MAFFT[1] (version 7.305b) to generate a multiple sequence alignment that included the HXB2 *pol* reference sequence (Genbank accession number K03455) as a scaffold for incomplete sequences. If the mean p-distance (proportion of nucleotide differences including mixtures) in the alignment with the HXB2 reference removed exceeded a cutoff of 5% and the total overlap was greater than 100 bases, the consensus sequence was flagged as the potential result of merging mislabeled samples. A total of 7 consensus sequences were flagged. When multiple sequences (*i.e.*, baseline and one or more failures) were available for a given patient, we used only the earliest failure sample for all cross-sectional analyses.

Amino acid polymorphisms were extracted from the sequence data by pairwise local alignment of each nucleotide sequence against the HXB2 reference. We used an implementation of the Gotoh algorithm in HyPhy with match/mismatch scores of 5 and -4 and gap open and extension penalties of 10 and 1, respectively. Any base insertions relative to the reference were discarded. The aligned sequences were translated into amino acids and recorded within the HXB2 coordinate system.

We obtained Stanford resistance predictions for the resulting consensus sequences using a custom Python script to automate transactions with the Stanford HIVdb Sierra web service[2] (algorithm version 8.1.1). The XML outputs generated by the Sierra web service were parsed using a Python script to extract drug-specific scores and to link the input sequences with their original labels. We calculated genotypic susceptibility scores (GSSs) in R by mapping Stanford resistance scores to five values according to the HIVdb algorithm version 7.0.1 ($[-\infty, 9] \rightarrow 1.0$, $[10, 14] \rightarrow 0.75$, $[15, 29] \rightarrow 0.5$, $[30, 59] \rightarrow 0.25$, $[60, \infty] \rightarrow 0$) to produce matrix S with rows corresponding to patients and columns for different drugs. A value of $S_{i,j} = 1.0$ indicates that patient i's infection is highly susceptible to drug j. Next, we extracted and sorted the drug exposure variables to generate a binary matrix E with the same dimensions as S and matching column order. Finally, we calculated GSS by summing across rows of the element-wise product of S and E.

HIV subtype classification and recombination detection was performed on the sequence data using both SCUEAL[3] and REGA[4]. The SCUEAL analysis was executed on our computing cluster with a high-sensitivity configuration of the genetic algorithm: a population size of 128 models; a stopping criterion of 100 generations without score improvement; a maximum of 5 recombination breakpoints; and a minimum recombination fragment length of 100 nucleotides. We used the 2011 HIV-1 *pol* reference alignment distributed with SCUEAL, which comprises 442 'pure' *pol* sequences from all HIV-1 group M, N, O and P subtypes and 39 circulating recombinant forms (CRFs). SCUEAL produces two levels of subtype and recombinant predictions: a low-level prediction that reports subtype reference assignments for each recombination fragment detected in the sequence, and a high-level summary of the subtype assignments. For example, a sequence with 2

recombination breakpoints (3 fragments) that are assigned respectively to subtypes A1, D and A1 is summarized as an A1/D recombinant; recombinants of three or more subtypes or sub-subtypes are labeled 'Complex'. We further reduced the high-level summaries by merging recombinants of sub-subtypes A1, A2, A3 and A4 as uniformly subtype A. Next, we re-categorized the sequences into subtypes A, C and D and A/D recombinants; any other subtypes, recombinants or CRFs were placed in an 'other' category. All subsequent references to SCUEAL subtype assignments will correspond to these simplified categories unless specified otherwise.

The REGA subtyping method was accessed using the web interface provided by the Stanford HIVdb database. Best subtype/recombinant assignments were extracted from REGA CSV outputs using an R script and reduced to high-level categories for comparison to the SCUEAL results. Subtype assignments for non-recombinant sequences were verified by phylogenetic reconstruction against the 2010 subtype reference set curated by the Los Alamos National Laboratory (LANL) HIV Sequence Database (http://www.hiv.lanl.gov/).

We used MAFFT to generate a multiple sequence alignment from all sequences classified as non-recombinant by SCUEAL, and manually refined the alignment using AliView[5]. For the purpose of visualizing subtypes, we reconstructed a phylogeny using FastTree2[6] under the general time-reversible model of nucleotide substitution. Bootstrap support values were estimated by the default Shimodaira-Hasegawa test[7] in FastTree2. Phylogenies were visualized and manually annotated using FigTree (A. Rambaut, http://tree.bio.ed.ac.uk/software/figtree).

Text S2: Bayesian network analysis

We used an implementation of the order Markov chain Monte Carlo (MCMC) method[8] in HyPhy[9] to perform a Bayesian network analysis. Bayesian networks are a class of machine learning methods, where the network is a probabilistic graphical model of the conditional dependencies among a number of variables[10]. Drug exposures and treatment failures were encoded as binary outcomes. Five drug exposure variables (ATV, DRV, SQV, IDV and ETR) were excluded for having an insufficient number of positives ($n \leq 5$). Region values were encoded for Fort Portal, Kampala, and Mbale; otherwise the record was excluded. Subtype values were encoded for the simplified categories (subtype A, C, and D, A/D recombinant, and 'other'). The final data set for the Bayesian network analysis comprised 1750 rows (observations) and 18 variables. We ran two replicate order-MCMC chain samples for a burn-in period of 10^5 steps, followed by 10^6 steps that were sampled at regular intervals of 2000 steps. Next, we compared the replicate chains to assess their convergence to the posterior distribution. An edge e_{ij} was included in the consensus Bayesian network if the sum of marginal posterior probabilities $P(e_{ij}) + P(e_{ji})$ exceeded a cutoff of 0.9. Edges were assigned the direction $i \to j$ if the proportion $P(e_{ij})/(P(e_{ij}) + P(e_{ji}))$ was greater than 0.8, $j \to i$ if it was lesser than 0.2, and left undirected otherwise.

Additional Figures

Figure S1: Statistical power to detect an association between first-line treatment failure and a specific subtype (X). We assume that 10^4 individuals have started first-line treatment, that the overall prevalence of treatment failure is $p = 5\%$, and that the prevalence of the subtype is $x = 20\%$. The subtype-specific prevalence of treatment failure was set to $q = pr/(1 - p(1 - r))$, where r is the expected odds ratio for a Fisher's exact test on the resulting 2×2 contingency table. We simulated sampling of individuals at random without replacement to obtain a target sample size N, and estimated power from the proportion of 1000 samples where Fisher's exact test rejected the null hypothesis given $r > 1$ (left). Next, we simulated weighted sampling where the proportion of treatment failures was modified to a specific percentage, given $N = 5000$, to reflect the impact of targeted sampling in a retrospective study (right). Note that the x-axis of the second plot was rescaled.

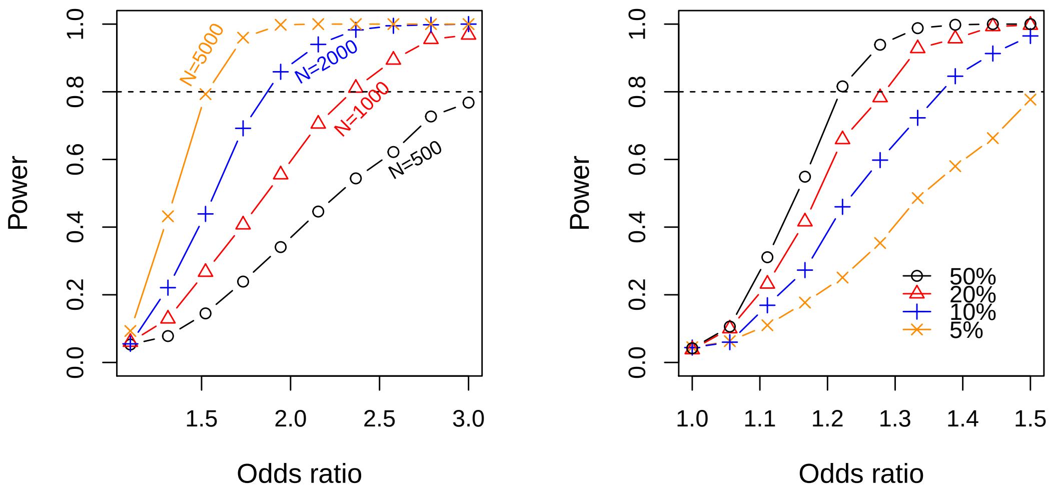

Figure S2: Phylogenetic tree of non-recombinant HIV-1 sequences annotated by subtype. The tree was arbitrarily rooted at the split between HIV-1 subtypes C and D. Simple subtype categories are indicated by colour (dark red = subtype A, blue = subtype C, green = subtype D). Orange branches indicate sequences in the 'other' category, including subtype U and circulating recombinant forms (CRFs), which are classified as non-recombinant because they were included in SCUEAL reference set. Two subtrees with a high proportion of subtype U are labeled on the plot; one of these subtrees (bootstrap support 81%) was placed between clades of subtype A (94%) and G (99%). We note that subtype G was recently proposed to be itself the product of a recombination involving a subtype A parental lineage[11]. Sequences within the subtype D clade that were classified as 'other' tended to be CRF10. The scale bar indicates the expected number of nucleotide substitutions per site.

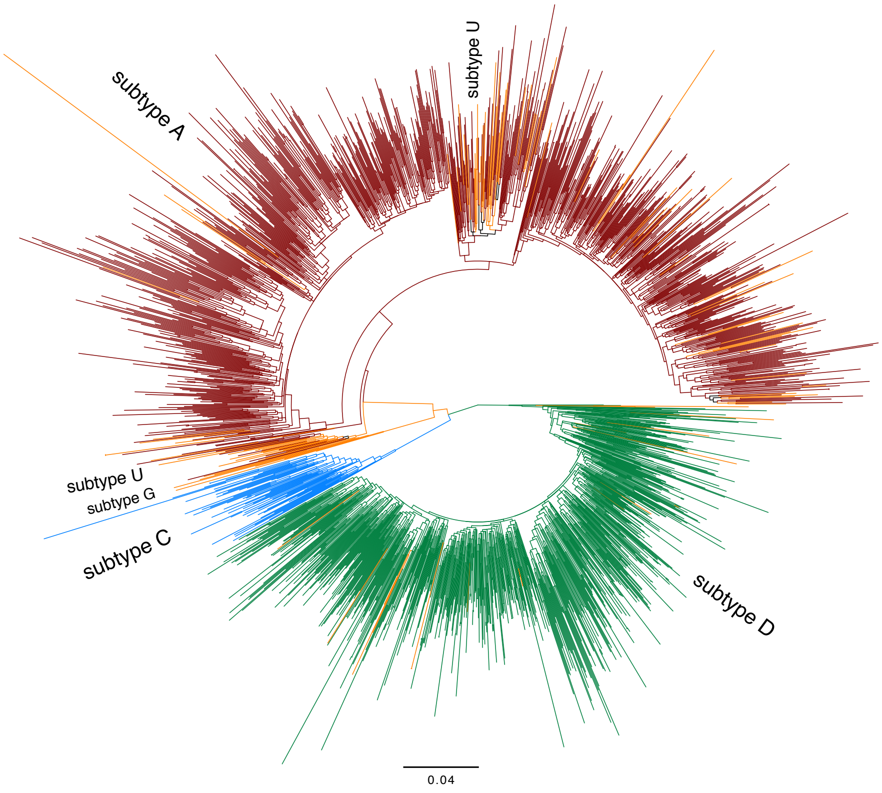

Figure S3: **Box-and-whisker plots of** $\log_{10}$ **plasma viral loads of baseline (open) and failure (shaded) samples among HIV subtypes.** Each box indicates the interquartile range (IQR) and is bisected by a line to indicate the median. The 'whiskers' extend to the furthest data points no further than 1.5 × IQR from the median; any points beyond these limits are classified outliers and represented by points on the plot. Sample sizes are annotated along the *x*-axis.

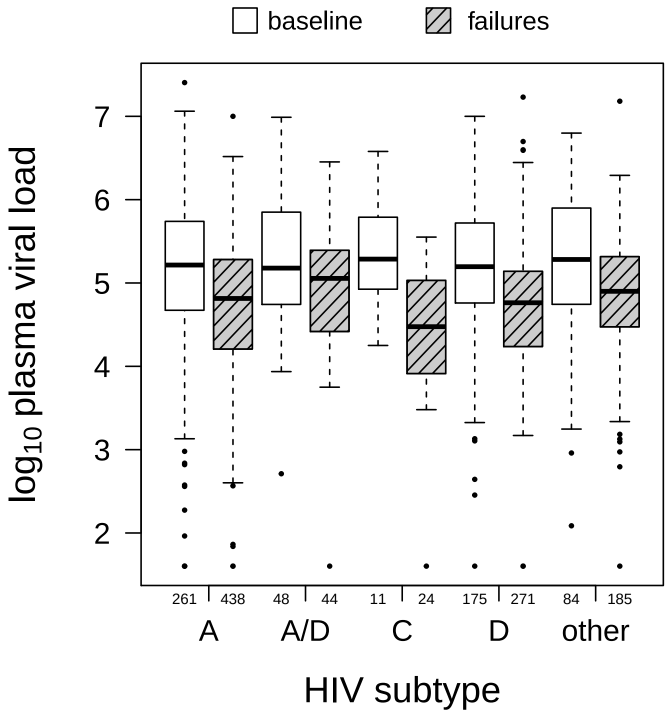

Figure S4: Mosaic plot of the contingency table of simple HIV-1 subtype categories against baseline and failure groups. Each box corresponds to a cell in the contingency table, scaled so that its area is proportional to the respective cell count. Boxes are coloured to indicate whether the observed count is significantly greater (blue) or lesser (red) than expected by chance. The *p*-value is obtained from a log-linear model analysis of the contingency table.

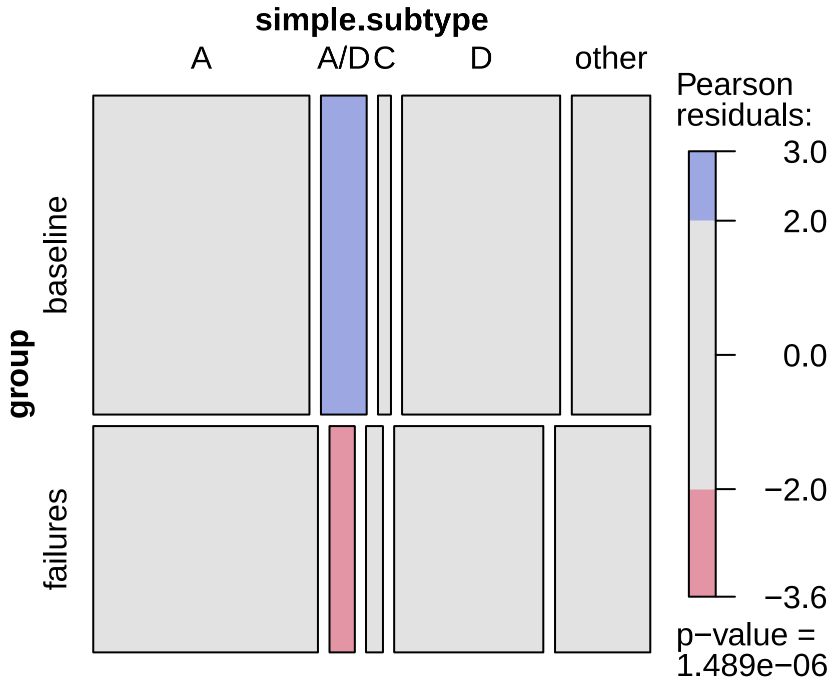

Figure S5: Visualization of breakpoint distribution among A/D recombinants, separated into baseline and failure sequences. Each line segment represents an HIV-1 *pol* sequence, with fragments coloured with respect to subtype A (red) or D (blue). We used agglomerative (complete-linkage) hierarchical clustering of binary distances to re-order the sequences with respect to their similarity in recombination breakpoints.

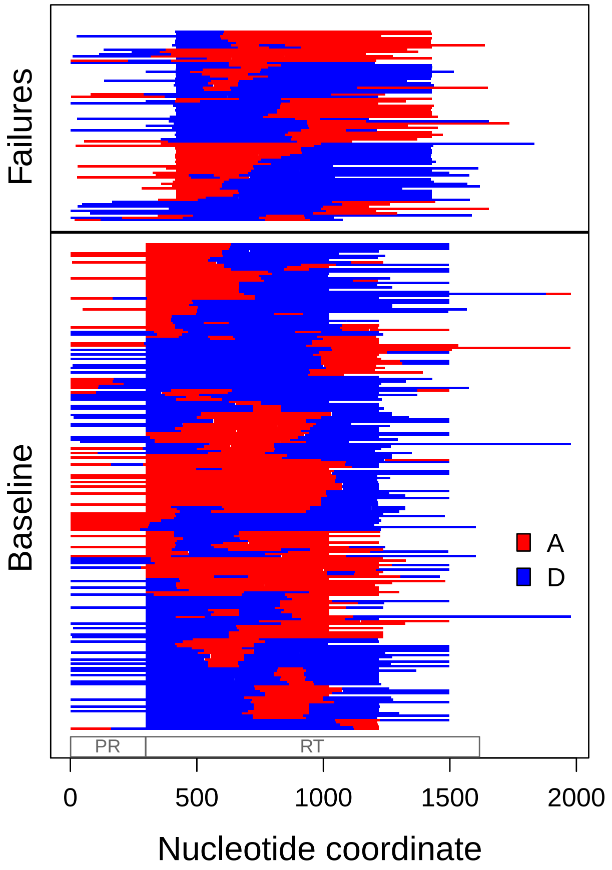

Additional Tables

Table S1: Summary table of databases from multiple study cohort and clinical sites in Uganda. Subtype classifications were generated using SCUEAL. The 'Other' category includes subtype C and circulating recombinant forms.

Source	Baseline	Failures	Subtype				
			A	D	A/D	C	Other
Joint Clinical Research Centre (JCRC)	160	1194	44%	29%	5%	3%	19%
Europe-Africa Research Network for Evaluation of Second-line Therapy (EARNEST)	0	356	43%	32%	5%	2%	18%
HIV-1 Genital Shedding (GS) study	81	0	43%	28%	11%	4%	10%
Monitoring Antiretroviral Resistance in Children (MARCH)	209	103	50%	28%	6%	2%	14%
Pan-African Studies to Evaluate Resistance (PASER)	518	71	44%	31%	8%	2%	15%
Stanford HIVdb (Uganda only)	1462	0	40%	32%	9%	3%	16%

Table S2: Concordance in subtype classification by SCUEAL and REGA. SCUEAL classifications were simplified and reduced to five categories (row labels). REGA (version 3.0) classifications were generated using the Stanford HIVdb web interface and reduced to the corresponding categories (column labels). 'NA' indicates that the REGA algorithm failed to produce a subtype classification for the sequence. Cohen's κ (a measure of agreement between two raters) was 0.716, where $\kappa = 1$ indicates complete agreement, and 0.937 if we excluded the 'other' categories.

	REGA					
SCUEAL	A	A/D	C	D	other	NA
A	**1692**	2	1	4	48	39
A/D	52	**154**	0	51	36	9
C	0	0	**113**	0	0	3
D	1	7	0	**1192**	24	30
other	357	80	14	86	144	15

References

1 Katoh K, Standley DM. MAFFT multiple sequence alignment software version 7: improvements in performance and usability. Mol Biol Evol. 2013 Apr;30(4):772–80.

2 Tang MW, Liu TF, Shafer RW. The HIVdb system for HIV-1 genotypic resistance interpretation. Intervirology. 2012;55(2):98–101.

3 Kosakovsky Pond SL, Posada D, Stawiski E, Chappey C, Poon AFY, Hughes G, et al. An evolutionary model-based algorithm for accurate phylogenetic breakpoint mapping and subtype prediction in HIV-1. PLoS Comput Biol. 2009 Nov;5(11):e1000581.

4 Pineda-Peña AC, Faria NR, Imbrechts S, Libin P, Abecasis AB, Deforche K, et al. Automated subtyping of HIV-1 genetic sequences for clinical and surveillance purposes: performance evaluation of the new REGA version 3 and seven other tools. Infect Genet Evol. 2013 Oct;19:337–48.

5 Larsson A. AliView: a fast and lightweight alignment viewer and editor for large datasets. Bioinformatics. 2014 Nov;30(22):3276–8.

6 Price MN, Dehal PS, Arkin AP. FastTree 2–approximately maximum-likelihood trees for large alignments. PLoS One. 2010 Mar;5(3):e9490.

7 Shimodaira H, Hasegawa M. Multiple comparisons of log-likelihoods with applications to phylogenetic inference. Molecular biology and evolution. 1999;16:1114–1116.

8 Friedman N, Koller D. Being Bayesian about network structure. A Bayesian approach to structure discovery in Bayesian networks. Machine learning. 2003;50(1-2):95–125.

9 Poon AFY, Lewis FI, Pond SLK, Frost SDW. An evolutionary-network model reveals stratified interactions in the V3 loop of the HIV-1 envelope. PLoS Comput Biol. 2007 Nov;3(11):e231.

10 Cooper GF, Herskovits E. A Bayesian method for the induction of probabilistic networks from data. Machine learning. 1992;9(4):309–347.

11 Abecasis AB, Lemey P, Vidal N, de Oliveira T, Peeters M, Camacho R, et al. Recombination confounds the early evolutionary history of human immunodeficiency virus type 1: subtype G is a circulating recombinant form. J Virol. 2007 Aug;81(16):8543–51.
